# RETRACTION: Effect of Laparoscopic Pancreaticoduodenectomy on the Incidence of Surgical‐Site Wound Infection: A Meta‐Analysis

**DOI:** 10.1111/iwj.70306

**Published:** 2025-03-06

## Abstract

**RETRACTION:** KongD.‐S. , ZhangH.‐L. , ZhaoX.‐L. , MengY. , ChaiW. , and WangZ.‐Y. , “Effect of Laparoscopic Pancreaticoduodenectomy on the Incidence of Surgical‐Site Wound Infection: A Meta‐Analysis,” International Wound Journal 20, no. 9 (2023): 3682–3689, 10.1111/iwj.14259.37277912 PMC10588349

The above article, published online on 05 June 2023, in Wiley Online Library (http://onlinelibrary.wiley.com/), has been retracted by agreement between the journal Editor in Chief, Professor Keith Harding; and John Wiley & Sons Ltd. Following an investigation by the publisher, all parties have concluded that this article was accepted solely on the basis of a compromised peer review process. The editors have therefore decided to retract the article. The authors did not respond to our notice regarding the retraction.

**RETRACTION:** D.‐S. Kong , H.‐L. Zhang , X.‐L. Zhao , Y. Meng , W. Chai , and Z.‐Y. Wang , “Effect of Laparoscopic Pancreaticoduodenectomy on the Incidence of Surgical‐Site Wound Infection: A Meta‐Analysis,” International Wound Journal 20, no. 9 (2023): 3682–3689, 10.1111/iwj.14259.37277912 PMC10588349

The above article, published online on 05 June 2023, in Wiley Online Library (http://onlinelibrary.wiley.com/), has been retracted by agreement between the journal Editor in Chief, Professor Keith Harding; and John Wiley & Sons Ltd. Following an investigation by the publisher, all parties have concluded that this article was accepted solely on the basis of a compromised peer review process. The editors have therefore decided to retract the article. The authors did not respond to our notice regarding the retraction.

